# Is there a better way to teach clinical risk assessment by addressing concepts, human ability and basic facts?

**DOI:** 10.1192/bjb.2025.14

**Published:** 2025-04

**Authors:** Sam Dearman, Sara Abdelhamid, Rooshan Khan

**Affiliations:** Consultant Psychiatrist, Carleton Clinic, Cumbria, Northumbria, Tyne and Wear NHS Foundation Trust, Carlisle, UK; CT3 Resident Doctor, Carleton Clinic, Cumbria, Northumbria, Tyne and Wear NHS Foundation Trust, Carlisle, UK; GP Resident Doctor, Carleton Clinic, Cumbria, Northumbria, Tyne and Wear NHS Foundation Trust, Carlisle, UK

Dear Editor

The assessment and management of the risk of harm, relating to a person with a mental illness is an extremely important and integral part of psychiatric practice and of safe decision-making in mental health services more broadly. In 2022, NICE moved away from the use of risk assessment tools and scales to predict future suicide or self-harm.^1^ It is also the case that empirical research cannot be relied upon to identify all risk factors.^2^ Risk training in mental health services typically, although not exclusively, focuses on suicide and self-harm, documentation formats and clinical enquiries such as holding plans and ideas of suicide. Despite this, our continued observation is that practitioners commonly use the term ‘no or low risk’ and base these complex judgements on reductionist factors such as the presence or absence of relevant thoughts disclosed.

We suggest that it is perhaps just as important to focus on training people to understand what a risk assessment is fundamentally attempting to achieve, what concepts this involves, what thinking errors and biases might be relevant, as well as what socio-demographic correlates are known in addition to the more familiar clinical enquiries. We devised a brief educational intervention for all staff in crisis services. The core components of this included learning that human beings are generally not very effective at determining expected outcomes, value and risk, as well as imparting knowledge of background rates in the general population, how to integrate socio-demographics and the more profound risk-increasing effect of past behaviour. In a pilot of the intervention we surveyed 16 staff prior and after the learning as to their self-evaluation using a five-point Likert scale of agreement with statements as to related knowledge and capabilities. The group included healthcare assistants, Band 5 nurses, as well as foundation year and general practitioner doctors in training.

We found that, overall, 69% (11/16) of the group reported an improvement in knowledge and capabilities, defined as a positive increase of one or more points of the scale. This trend continued across all domains with 62.5% (10/16) reporting increased competence in assessment, 62.5% (10/16) reporting increased competence in communicating risk, 69% (11/16) reporting increased knowledge of risk-increasing factors, 69% (11/16) reporting an increased ability to differentiate between lower and higher levels of risk and 69% (11/16) reporting increased understanding of the concept of risk. This trend continued when the data were sub-analysed by profession.

The process of assessing risk in relation to complex human behaviour is necessarily multifactorial but is also fundamentally influenced by an individual's concept of risk itself as well as what the basis of comparison is. We believe the findings from our pilot intervention suggest that further evaluation of an approach that includes these other factors warrants further exploration.
